# Dicarbon­yl(hexa­methyl­ene-1,3,5,7-tetra­mine-κ*N* ^1^)(η^5^-penta­methyl­cyclo­penta­dien­yl)iron(II) tetra­fluoridoborate

**DOI:** 10.1107/S1600536812026360

**Published:** 2012-06-16

**Authors:** Cyprian M. M’thiruaine, Holger B. Friedrich, Evans O. Changamu, Manuel A. Fernandes

**Affiliations:** a School of Chemistry, University of KwaZulu-Natal, Private Bag X54001, Durban 4000, South Africa; b Chemistry Department, Kenyatta University, PO Box 43844, Nairobi, Kenya; c Molecular Sciences Institute, School of Chemistry, University of the Witwatersrand, PO Wits, 2050 Johannesburg, South Africa

## Abstract

In the title compound, [Fe(C_10_H_15_)(C_6_H_12_N_4_)(CO)_2_]BF_4_, the arrangement around the Fe^II^ atom corresponds to a three-legged piano stool. The penta­methyl­cyclo­penta­dienyl (Cp*) ligand occupies three coordination sites, while two CO ligands and one N atom of the hexa­methyl­ene­tetra­mine ligand occupy the remaining coordination sites, completing a pseudo-octahedral geometry. Both the complex cation and the BF_4_^−^ anion reside on crystallographic mirror planes. The Fe—N bond length is 2.069 (2) and the Fe—Cp*(centroid) distance is 1.7452 (3) Å.

## Related literature

For the synthesis of the title compound and structure of the dinuclear compound [Fe_2_(η^5^-C_5_H_5_)_2_{N_4_(CH_2_)_6_}(CO)_4_](BF_4_)_2_ see: M’thiruaine *et al.* (2012**a* ▶)*. For other related compounds, see: Allan *et al.* (1970 ▶); Darensbourg *et al.* (2003 ▶); Lu *et al.* (2004 ▶); Matos & Verkade (2003 ▶); M’thiruaine *et al.* (2012*b* ▶); Shafiq *et al.* (2000 ▶). For mol­ecular structures of other metal complexes of hexa­methyl­ene­tetra­mine, see: Zheng *et al.* (2008 ▶); Xue *et al.* (2009 ▶). For applications of hexa­methyl­ene­tetra­mine, see: Greenwood (1981 ▶); Strom & Jun (1986 ▶); Garcia *et al.* (2010 ▶).
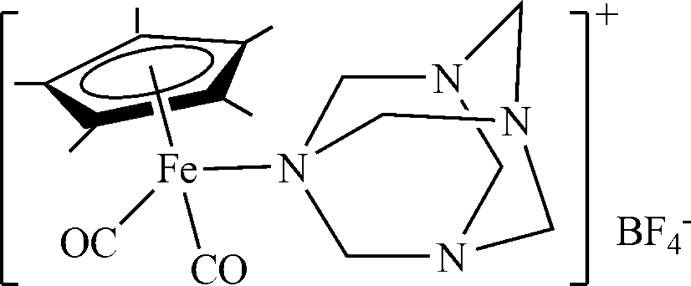


## Experimental

### 

#### Crystal data

[Fe(C_10_H_15_)(C_6_H_12_N_4_)(CO)_2_]BF_4_
*M* *_r_* = 474.10
Orthorhombic, 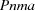

*a* = 13.8388 (6) Å
*b* = 9.1771 (4) Å
*c* = 16.4365 (8) Å
*V* = 2087.44 (16) Å^3^
*Z* = 4
Mo *K*α radiation
μ = 0.78 mm^−1^
*T* = 173 K
0.40 × 0.40 × 0.40 mm

#### Data collection

Bruker APEXII CCD diffractometer
Absorption correction: integration (*SADABS*; Bruker, 2005 ▶) *T*_min_ = 0.746, *T*_max_ = 0.746
16663 measured reflections
2671 independent reflections
2065 reflections with *I* > 2σ(*I*)
*R*_int_ = 0.050

#### Refinement

*R*[*F* ^2^ > 2σ(*F* ^2^)] = 0.033
*wR*(*F* ^2^) = 0.094
*S* = 1.04
2671 reflections
153 parameters
H-atom parameters constrained
Δρ_max_ = 0.56 e Å^−3^
Δρ_min_ = −0.55 e Å^−3^

### 

Data collection: *APEX2* (Bruker, 2005 ▶); cell refinement: *SAINT-Plus* (Bruker, 2005 ▶); data reduction: *SAINT-Plus* and *XPREP* (Bruker, 2005 ▶); program(s) used to solve structure: *SHELXS97* (Sheldrick, 2008 ▶); program(s) used to refine structure: *SHELXL97* (Sheldrick, 2008 ▶); molecular graphics: *ORTEP-3* Farrugia (1997 ▶); software used to prepare material for publication: *SHELXL97* and *PLATON* (Spek, 2009 ▶).

## Supplementary Material

Crystal structure: contains datablock(s) I, global. DOI: 10.1107/S1600536812026360/fj2565sup1.cif

Structure factors: contains datablock(s) I. DOI: 10.1107/S1600536812026360/fj2565Isup2.hkl

Additional supplementary materials:  crystallographic information; 3D view; checkCIF report

## Figures and Tables

**Fig. 1.**
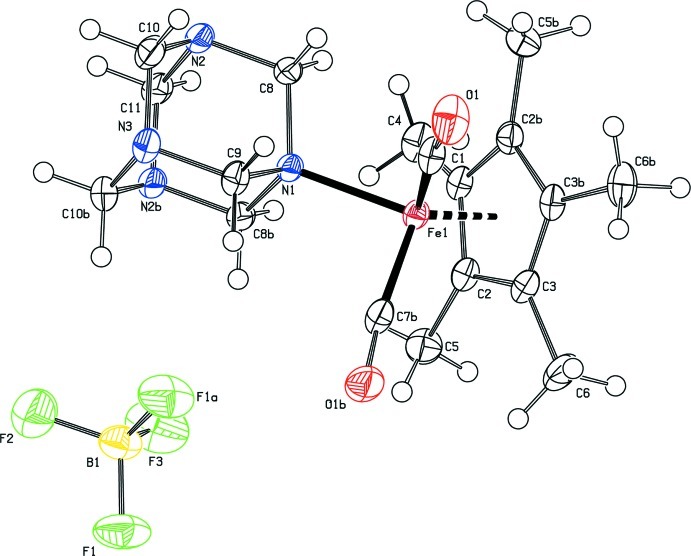
The molecular structure of the title complex with the atom labeling scheme. Ellipsoids are drawn at 50% probability level.

## supplementary crystallographic information

### Comment

Hexamethylenetetramine has been used as an antibacterial agent in the treatment of urinary tract infections for several years (Greenwood, 1981; Strom and Jun, 1986) and recently it has found application as a base catalyst to control the porosity and pores size of resorcinol furaldehyde cryogels synthesized in *t*-butanol (Garcia *et al.*, 2010). A number of structures of metal-coordinated hexamethylenetetramine have been reported (Lu *et al.*, 2004; Zheng *et al.*, 2008; Xue *et al.*, 2009) but very few in which hexamethylenetetramine is directly coordinated to iron are known (Allan *et al.*, 1970).

The title compound was obtained as part of our ongoing investigation of the reactions of substitutionally unsaturated metal complexes with nitrogen donor ligands (M'thiruaine *et al.*, 2012*a*; M'thiruaine *et al.*, 2012*b*). The synthesis and characterization data was previously reported by us, but its crystal structure is not known. To the best of our knowledge the structure of the title compound is the first of the hexamethylenetetramine complex containing the Cp*(CO)_2_Fe moiety to be reported. It exhibits a typical three legged piano stool structure with Fe^II^ coordinated by hexamethylenetetramine through the nitrogen atom, in which the coordination geometry around Fe can be described as distorted octahedral with three sites occupied by the η^5^-pentamethylcyclopentadienyl ligand, while the two CO ligands and hexamethylenetetramine occupy the remaining three sites to complete the octahedron (Fig 1). Its structure is similar to those of the anionic 1,3,5-triaza-7-phosphaadamantane (PTA) complexes [CpFe(CN)_2_(PTA)]^-^, [CpFe(CN)_2_(PTAH)]^-^ (Darensbourg *et al.*, 2003) and that of neutral [CpW(CO)_2_(PTA)H] (Shafiq *et al.*, 2000).

Both the iron complex and the BF_4_^-^ anion crystallize on mirror planes at b= 0.25 and b=0.75 in the crystal structure of the title compound. In the case of the iron complex, the mirror plane goes through the iron atom and bisects the pentadienyl ligand and tertraamine equally, with the carbonyl atoms being located on either side of the mirror plane. In the case of the BF_4_^-^ anion, the boron atom and two of the flourine atoms (F2 and F3) are located on the mirror plane, while the remaining flourine atoms are located at either side of the mirror. Consequently, the asymmetric unit of the title compound contains half a monocationic molecule and half a counter-anion. From a molecular structure point of view, the Fe—N bond was found to have a length of 2.069 (2) Å, which is slightly shorter than the 2.0817 (17) and 2.0858 (18) Å distances reported for the dinuclear complex [{Cp(CO)_2_Fe}_2_{N_4_(CH_2_)_6_}]^2+^ (M'thiruaine *et al.*, 2012*a*) and 2.092 (4) Å reported for [(CO)_4_Fe{N_2_(CH_2_)_6_}] (Matos and Verkade, 2003).

### Experimental

The title compound was prepared according to a reported procedure (M'thiruaine *et al.* 2012*a*) and crystals were grown by layering a concentrated solution of the compound in acetone with Et_2_O and the mixture kept undisturbed in the dark for four weeks.

### Refinement

Non-hydrogen atoms were first refined isotropically followed by anisotropic refinement by full matrix least-square calculations on *F^2^* using *SHEXTL*. Hydrogen atoms were first located in the difference map then positioned geometrically and allowed to ride on their respective parent atoms.

### Crystal data

**Table:**

[Fe(C_10_H_15_)(C_6_H_12_N_4_)(CO)_2_]BF_4_	*F*(000) = 984
*M**_r_* = 474.10	*D*_x_ = 1.509 Mg m^−^^3^
Orthorhombic, *P**n**m**a*	Mo *K*α radiation, λ = 0.71073 Å
Hall symbol: -P 2ac 2n	Cell parameters from 4126 reflections
*a* = 13.8388 (6) Å	θ = 2.5–26.9°
*b* = 9.1771 (4) Å	µ = 0.78 mm^−^^1^
*c* = 16.4365 (8) Å	*T* = 173 K
*V* = 2087.44 (16) Å^3^	Block, brown
*Z* = 4	0.40 × 0.40 × 0.40 mm

### Data collection

**Table:**

Bruker APEXII CCD diffractometer	2671 independent reflections
Radiation source: fine-focus sealed tube	2065 reflections with *I* > 2σ(*I*)
Graphite monochromator	*R*_int_ = 0.050
φ and ω scans	θ_max_ = 28.0°, θ_min_ = 1.9°
Absorption correction: integration (*SADABS*; Bruker, 2005)	*h* = −17→18
*T*_min_ = 0.746, *T*_max_ = 0.746	*k* = −12→12
16663 measured reflections	*l* = −14→21

### Refinement

**Table:**

Refinement on *F*^2^	Primary atom site location: structure-invariant direct methods
Least-squares matrix: full	Secondary atom site location: difference Fourier map
*R*[*F*^2^ > 2σ(*F*^2^)] = 0.033	Hydrogen site location: inferred from neighbouring sites
*wR*(*F*^2^) = 0.094	H-atom parameters constrained
*S* = 1.04	*w* = 1/[σ^2^(*F*_o_^2^) + (0.0514*P*)^2^] where *P* = (*F*_o_^2^ + 2*F*_c_^2^)/3
2671 reflections	(Δ/σ)_max_ = 0.008
153 parameters	Δρ_max_ = 0.56 e Å^−^^3^
0 restraints	Δρ_min_ = −0.55 e Å^−^^3^

### Special details

**Table:**

Experimental. face indexed absorption corrections carried out with XPREP; Bruker, 2005)
Geometry. All e.s.d.'s (except the e.s.d. in the dihedral angle between two l.s. planes) are estimated using the full covariance matrix. The cell e.s.d.'s are taken into account individually in the estimation of e.s.d.'s in distances, angles and torsion angles; correlations between e.s.d.'s in cell parameters are only used when they are defined by crystal symmetry. An approximate (isotropic) treatment of cell e.s.d.'s is used for estimating e.s.d.'s involving l.s. planes.
Refinement. Refinement of *F^2^* against ALL reflections. The weighted *R*-factor *wR* and goodness of fit *S* are based on *F^2^*, conventional *R*-factors *R* are based on *F*, with *F* set to zero for negative *F^2^*. The threshold expression of *F^2^* > σ(*F^2^*) is used only for calculating *R*-factors(gt) *etc*. and is not relevant to the choice of reflections for refinement. *R*-factors based on *F^2^* are statistically about twice as large as those based on *F*, and *R*- factors based on ALL data will be even larger.

### Fractional atomic coordinates and isotropic or equivalent isotropic displacement parameters (Å^2^)

**Table:**

	*x*	*y*	*z*	*U*_iso_*/*U*_eq_	Occ. (<1)
C1	−0.07473 (16)	0.7500	0.29615 (16)	0.0216 (5)	
C2	−0.04858 (13)	0.8758 (2)	0.25029 (10)	0.0225 (4)	
C3	−0.01373 (12)	0.82727 (19)	0.17274 (10)	0.0239 (4)	
C4	−0.13130 (18)	0.7500	0.37414 (16)	0.0286 (6)	
H4A	−0.1161	0.6660	0.4052	0.043*	0.50
H4B	−0.1985	0.7500	0.3629	0.043*	
H4C	−0.1161	0.8340	0.4052	0.043*	0.50
C5	−0.06466 (14)	1.0313 (2)	0.27470 (13)	0.0304 (4)	
H5A	−0.1229	1.0685	0.2478	0.046*	
H5B	−0.0088	1.0900	0.2584	0.046*	
H5C	−0.0728	1.0370	0.3338	0.046*	
C6	0.00822 (15)	0.9225 (2)	0.10053 (12)	0.0351 (5)	
H6A	0.0601	0.8783	0.0683	0.053*	
H6B	0.0287	1.0190	0.1194	0.053*	
H6C	−0.0499	0.9324	0.0668	0.053*	
C7	0.15593 (13)	0.6020 (2)	0.23589 (11)	0.0263 (4)	
C8	0.09551 (13)	0.61629 (18)	0.43011 (11)	0.0220 (4)	
H8A	0.0240	0.6120	0.4298	0.026*	
H8B	0.1200	0.5280	0.4023	0.026*	
C9	0.23775 (17)	0.7500	0.38815 (15)	0.0238 (5)	
H9A	0.2629	0.6628	0.3597	0.029*	0.50
H9B	0.2629	0.8372	0.3597	0.029*	0.50
C10	0.23601 (14)	0.61969 (19)	0.51331 (11)	0.0295 (4)	
H10A	0.2601	0.6186	0.5700	0.035*	
H10B	0.2606	0.5313	0.4857	0.035*	
C11	0.0944 (2)	0.7500	0.55398 (16)	0.0278 (6)	
H11A	0.1160	0.7500	0.6114	0.033*	
H11B	0.0228	0.7500	0.5537	0.033*	
B1	0.2554 (3)	0.2500	0.4092 (2)	0.0361 (8)	
F1	0.28284 (11)	0.12563 (13)	0.36756 (8)	0.0570 (4)	
F2	0.29634 (17)	0.2500	0.48627 (13)	0.0660 (6)	
F3	0.15679 (15)	0.2500	0.41946 (15)	0.0703 (7)	
Fe1	0.07875 (2)	0.7500	0.26471 (2)	0.01882 (12)	
N1	0.12822 (14)	0.7500	0.38349 (12)	0.0187 (4)	
N2	0.12944 (12)	0.61662 (15)	0.51387 (9)	0.0253 (3)	
N3	0.27327 (16)	0.7500	0.47139 (13)	0.0269 (5)	
O1	0.19858 (10)	0.50678 (17)	0.21102 (9)	0.0410 (4)	

### Atomic displacement parameters (Å^2^)

**Table:**

	*U*^11^	*U*^22^	*U*^33^	*U*^12^	*U*^13^	*U*^23^
C1	0.0185 (12)	0.0302 (13)	0.0161 (12)	0.000	−0.0015 (9)	0.000
C2	0.0204 (9)	0.0277 (9)	0.0194 (9)	0.0024 (7)	−0.0024 (7)	0.0002 (7)
C3	0.0224 (9)	0.0318 (10)	0.0174 (9)	0.0007 (7)	−0.0027 (7)	0.0037 (7)
C4	0.0205 (13)	0.0430 (16)	0.0223 (14)	0.000	0.0035 (11)	0.000
C5	0.0316 (11)	0.0271 (9)	0.0326 (12)	0.0070 (8)	−0.0011 (8)	−0.0017 (8)
C6	0.0375 (11)	0.0463 (12)	0.0216 (10)	−0.0025 (9)	−0.0019 (8)	0.0118 (9)
C7	0.0247 (10)	0.0361 (10)	0.0182 (9)	0.0006 (8)	−0.0031 (7)	−0.0028 (8)
C8	0.0296 (10)	0.0189 (8)	0.0177 (9)	−0.0013 (7)	−0.0010 (7)	0.0008 (7)
C9	0.0197 (12)	0.0320 (13)	0.0198 (13)	0.000	−0.0020 (10)	0.000
C10	0.0377 (11)	0.0289 (10)	0.0219 (10)	0.0049 (8)	−0.0073 (8)	0.0024 (8)
C11	0.0410 (16)	0.0265 (13)	0.0158 (13)	0.000	0.0015 (11)	0.000
B1	0.045 (2)	0.0277 (15)	0.036 (2)	0.000	0.0101 (15)	0.000
F1	0.0836 (11)	0.0364 (7)	0.0509 (9)	0.0058 (7)	0.0261 (7)	−0.0094 (6)
F2	0.0916 (17)	0.0588 (13)	0.0476 (13)	0.000	−0.0102 (12)	0.000
F3	0.0478 (13)	0.0654 (14)	0.0979 (19)	0.000	0.0209 (12)	0.000
Fe1	0.0191 (2)	0.02309 (19)	0.01423 (19)	0.000	0.00091 (13)	0.000
N1	0.0216 (10)	0.0195 (10)	0.0151 (10)	0.000	0.0006 (8)	0.000
N2	0.0361 (9)	0.0220 (7)	0.0178 (8)	0.0003 (6)	−0.0030 (6)	0.0009 (6)
N3	0.0295 (12)	0.0298 (11)	0.0213 (11)	0.000	−0.0066 (9)	0.000
O1	0.0396 (8)	0.0480 (9)	0.0354 (8)	0.0173 (7)	−0.0028 (7)	−0.0147 (7)

### Geometric parameters (Å, º)

**Table:**

C1—C2	1.425 (2)	C8—H8B	0.9900
C1—C2^i^	1.425 (2)	C9—N3	1.454 (3)
C1—C4	1.502 (3)	C9—N1	1.518 (3)
C1—Fe1	2.186 (2)	C9—H9A	0.9900
C2—C3	1.434 (2)	C9—H9B	0.9900
C2—C5	1.499 (2)	C10—N3	1.473 (2)
C2—Fe1	2.1199 (18)	C10—N2	1.475 (3)
C3—C3^i^	1.418 (3)	C10—H10A	0.9900
C3—C6	1.505 (2)	C10—H10B	0.9900
C3—Fe1	2.1038 (17)	C11—N2	1.473 (2)
C4—H4A	0.9482	C11—N2^i^	1.473 (2)
C4—H4B	0.9477	C11—H11A	0.9900
C4—H4C	0.9482	C11—H11B	0.9900
C5—H5A	0.9800	B1—F3	1.375 (4)
C5—H5B	0.9800	B1—F1^ii^	1.384 (2)
C5—H5C	0.9800	B1—F1	1.384 (2)
C6—H6A	0.9800	B1—F2	1.388 (4)
C6—H6B	0.9800	Fe1—C7^i^	1.791 (2)
C6—H6C	0.9800	Fe1—N1	2.069 (2)
C7—O1	1.131 (2)	Fe1—C3^i^	2.1038 (17)
C7—Fe1	1.791 (2)	Fe1—C2^i^	2.1199 (18)
C8—N2	1.455 (2)	N1—C8^i^	1.516 (2)
C8—N1	1.516 (2)	N3—C10^i^	1.473 (2)
C8—H8A	0.9900		
			
C2—C1—C2^i^	108.2 (2)	H10A—C10—H10B	108.0
C2—C1—C4	125.70 (11)	N2—C11—N2^i^	112.4 (2)
C2^i^—C1—C4	125.70 (11)	N2—C11—H11A	109.1
C2—C1—Fe1	68.18 (12)	N2^i^—C11—H11A	109.1
C2^i^—C1—Fe1	68.18 (12)	N2—C11—H11B	109.1
C4—C1—Fe1	135.09 (18)	N2^i^—C11—H11B	109.1
C1—C2—C3	107.69 (16)	H11A—C11—H11B	107.8
C1—C2—C5	126.29 (17)	F3—B1—F1^ii^	109.5 (2)
C3—C2—C5	125.70 (16)	F3—B1—F1	109.5 (2)
C1—C2—Fe1	73.19 (12)	F1^ii^—B1—F1	111.1 (3)
C3—C2—Fe1	69.55 (10)	F3—B1—F2	107.1 (3)
C5—C2—Fe1	127.73 (13)	F1^ii^—B1—F2	109.8 (2)
C3^i^—C3—C2	108.09 (10)	F1—B1—F2	109.8 (2)
C3^i^—C3—C6	125.52 (11)	C7—Fe1—C7^i^	98.58 (12)
C2—C3—C6	126.05 (16)	C7—Fe1—N1	93.00 (7)
C3^i^—C3—Fe1	70.30 (5)	C7^i^—Fe1—N1	93.00 (7)
C2—C3—Fe1	70.76 (10)	C7—Fe1—C3^i^	85.25 (8)
C6—C3—Fe1	129.76 (13)	C7^i^—Fe1—C3^i^	115.35 (8)
C1—C4—H4A	110.1	N1—Fe1—C3^i^	151.56 (6)
C1—C4—H4B	110.2	C7—Fe1—C3	115.35 (8)
H4A—C4—H4B	108.8	C7^i^—Fe1—C3	85.25 (8)
C1—C4—H4C	110.1	N1—Fe1—C3	151.56 (6)
H4A—C4—H4C	108.8	C3^i^—Fe1—C3	39.40 (10)
H4B—C4—H4C	108.8	C7—Fe1—C2^i^	93.05 (8)
C2—C5—H5A	109.5	C7^i^—Fe1—C2^i^	151.50 (8)
C2—C5—H5B	109.5	N1—Fe1—C2^i^	112.37 (7)
H5A—C5—H5B	109.5	C3^i^—Fe1—C2^i^	39.69 (7)
C2—C5—H5C	109.5	C3—Fe1—C2^i^	66.27 (7)
H5A—C5—H5C	109.5	C7—Fe1—C2	151.50 (8)
H5B—C5—H5C	109.5	C7^i^—Fe1—C2	93.05 (8)
C3—C6—H6A	109.5	N1—Fe1—C2	112.37 (7)
C3—C6—H6B	109.5	C3^i^—Fe1—C2	66.27 (7)
H6A—C6—H6B	109.5	C3—Fe1—C2	39.69 (7)
C3—C6—H6C	109.5	C2^i^—Fe1—C2	65.99 (10)
H6A—C6—H6C	109.5	C7—Fe1—C1	129.93 (6)
H6B—C6—H6C	109.5	C7^i^—Fe1—C1	129.93 (6)
O1—C7—Fe1	172.94 (17)	N1—Fe1—C1	95.65 (9)
N2—C8—N1	112.36 (14)	C3^i^—Fe1—C1	65.09 (8)
N2—C8—H8A	109.1	C3—Fe1—C1	65.09 (8)
N1—C8—H8A	109.1	C2^i^—Fe1—C1	38.62 (6)
N2—C8—H8B	109.1	C2—Fe1—C1	38.62 (6)
N1—C8—H8B	109.1	C8^i^—N1—C8	108.09 (18)
H8A—C8—H8B	107.9	C8^i^—N1—C9	105.83 (12)
N3—C9—N1	112.7 (2)	C8—N1—C9	105.83 (12)
N3—C9—H9A	109.1	C8^i^—N1—Fe1	112.22 (10)
N1—C9—H9A	109.1	C8—N1—Fe1	112.22 (10)
N3—C9—H9B	109.1	C9—N1—Fe1	112.21 (14)
N1—C9—H9B	109.1	C8—N2—C11	108.61 (16)
H9A—C9—H9B	107.8	C8—N2—C10	108.47 (14)
N3—C10—N2	111.61 (15)	C11—N2—C10	108.45 (16)
N3—C10—H10A	109.3	C9—N3—C10^i^	108.77 (13)
N2—C10—H10A	109.3	C9—N3—C10	108.77 (13)
N3—C10—H10B	109.3	C10^i^—N3—C10	108.5 (2)
N2—C10—H10B	109.3		
			
C2^i^—C1—C2—C3	4.9 (3)	C2^i^—C1—Fe1—C7	−20.56 (17)
C4—C1—C2—C3	−168.0 (2)	C4—C1—Fe1—C7	98.70 (10)
Fe1—C1—C2—C3	61.35 (13)	C2—C1—Fe1—C7^i^	20.56 (17)
C2^i^—C1—C2—C5	178.72 (12)	C2^i^—C1—Fe1—C7^i^	142.04 (11)
C4—C1—C2—C5	5.8 (4)	C4—C1—Fe1—C7^i^	−98.70 (10)
Fe1—C1—C2—C5	−124.84 (19)	C2—C1—Fe1—N1	119.26 (11)
C2^i^—C1—C2—Fe1	−56.44 (17)	C2^i^—C1—Fe1—N1	−119.26 (11)
C4—C1—C2—Fe1	130.7 (2)	C4—C1—Fe1—N1	0.0
C1—C2—C3—C3^i^	−3.03 (16)	C2—C1—Fe1—C3^i^	−82.56 (13)
C5—C2—C3—C3^i^	−176.89 (15)	C2^i^—C1—Fe1—C3^i^	38.92 (11)
Fe1—C2—C3—C3^i^	60.69 (12)	C4—C1—Fe1—C3^i^	158.18 (5)
C1—C2—C3—C6	170.56 (18)	C2—C1—Fe1—C3	−38.92 (11)
C5—C2—C3—C6	−3.3 (3)	C2^i^—C1—Fe1—C3	82.56 (13)
Fe1—C2—C3—C6	−125.73 (18)	C4—C1—Fe1—C3	−158.18 (5)
C1—C2—C3—Fe1	−63.71 (14)	C2—C1—Fe1—C2^i^	−121.5 (2)
C5—C2—C3—Fe1	122.43 (19)	C4—C1—Fe1—C2^i^	119.26 (11)
C3^i^—C3—Fe1—C7	43.83 (7)	C2^i^—C1—Fe1—C2	121.5 (2)
C2—C3—Fe1—C7	162.15 (11)	C4—C1—Fe1—C2	−119.26 (11)
C6—C3—Fe1—C7	−76.48 (19)	N2—C8—N1—C8^i^	−55.2 (2)
C3^i^—C3—Fe1—C7^i^	141.10 (6)	N2—C8—N1—C9	57.82 (19)
C2—C3—Fe1—C7^i^	−100.59 (12)	N2—C8—N1—Fe1	−179.48 (11)
C6—C3—Fe1—C7^i^	20.78 (18)	N3—C9—N1—C8^i^	57.29 (11)
C3^i^—C3—Fe1—N1	−131.39 (13)	N3—C9—N1—C8	−57.29 (11)
C2—C3—Fe1—N1	−13.07 (18)	N3—C9—N1—Fe1	180.0
C6—C3—Fe1—N1	108.29 (19)	C7—Fe1—N1—C8^i^	168.40 (13)
C2—C3—Fe1—C3^i^	118.32 (9)	C7^i^—Fe1—N1—C8^i^	69.64 (13)
C6—C3—Fe1—C3^i^	−120.32 (16)	C3^i^—Fe1—N1—C8^i^	−106.04 (13)
C3^i^—C3—Fe1—C2^i^	−37.89 (6)	C3—Fe1—N1—C8^i^	−15.9 (2)
C2—C3—Fe1—C2^i^	80.43 (14)	C2^i^—Fe1—N1—C8^i^	−97.06 (12)
C6—C3—Fe1—C2^i^	−158.20 (19)	C2—Fe1—N1—C8^i^	−24.90 (14)
C3^i^—C3—Fe1—C2	−118.32 (9)	C1—Fe1—N1—C8^i^	−60.98 (12)
C6—C3—Fe1—C2	121.4 (2)	C7—Fe1—N1—C8	−69.64 (13)
C3^i^—C3—Fe1—C1	−80.43 (9)	C7^i^—Fe1—N1—C8	−168.40 (13)
C2—C3—Fe1—C1	37.89 (9)	C3^i^—Fe1—N1—C8	15.9 (2)
C6—C3—Fe1—C1	159.25 (19)	C3—Fe1—N1—C8	106.04 (13)
C1—C2—Fe1—C7	81.4 (2)	C2^i^—Fe1—N1—C8	24.90 (14)
C3—C2—Fe1—C7	−35.5 (2)	C2—Fe1—N1—C8	97.06 (12)
C5—C2—Fe1—C7	−155.42 (17)	C1—Fe1—N1—C8	60.98 (12)
C1—C2—Fe1—C7^i^	−164.35 (13)	C7—Fe1—N1—C9	49.38 (6)
C3—C2—Fe1—C7^i^	78.81 (12)	C7^i^—Fe1—N1—C9	−49.38 (6)
C5—C2—Fe1—C7^i^	−41.12 (17)	C3^i^—Fe1—N1—C9	134.94 (12)
C1—C2—Fe1—N1	−69.85 (12)	C3—Fe1—N1—C9	−134.94 (12)
C3—C2—Fe1—N1	173.31 (9)	C2^i^—Fe1—N1—C9	143.92 (6)
C5—C2—Fe1—N1	53.38 (17)	C2—Fe1—N1—C9	−143.92 (6)
C1—C2—Fe1—C3^i^	79.22 (12)	C1—Fe1—N1—C9	180.0
C3—C2—Fe1—C3^i^	−37.62 (11)	N1—C8—N2—C11	57.6 (2)
C5—C2—Fe1—C3^i^	−157.54 (18)	N1—C8—N2—C10	−60.06 (18)
C1—C2—Fe1—C3	116.84 (16)	N2^i^—C11—N2—C8	−60.0 (3)
C5—C2—Fe1—C3	−119.9 (2)	N2^i^—C11—N2—C10	57.7 (2)
C1—C2—Fe1—C2^i^	35.65 (13)	N3—C10—N2—C8	59.72 (18)
C3—C2—Fe1—C2^i^	−81.19 (10)	N3—C10—N2—C11	−58.1 (2)
C5—C2—Fe1—C2^i^	158.88 (14)	N1—C9—N3—C10^i^	−59.01 (14)
C3—C2—Fe1—C1	−116.84 (16)	N1—C9—N3—C10	59.01 (14)
C5—C2—Fe1—C1	123.2 (2)	N2—C10—N3—C9	−59.2 (2)
C2—C1—Fe1—C7	−142.04 (11)	N2—C10—N3—C10^i^	59.0 (2)

Symmetry codes: (i) *x*, −*y*+3/2, *z*; (ii) *x*, −*y*+1/2, *z*.
